# Bouveret's syndrome as an unusual cause of gastric outlet obstruction: a case report

**DOI:** 10.1186/1752-1947-1-73

**Published:** 2007-08-30

**Authors:** Deepak Joshi, Ali Vosough, Tom M Raymond, Chris Fox, Arun Dhiman

**Affiliations:** 1 Department of Gastroenterology, William Harvey Hospital, Ashford, Kent, UK; 2 Department of Surgery, William Harvey Hospital, Ashford, Kent, UK

## Abstract

An 83 year old caucasian gentleman presented with vomiting and left sided abdominal pain. A subsequent upper GI endoscopy demonstrated a large smooth mass impacted within the duodenum. A cholecysto-duodenal fistula was discovered at laparotomy, with a large gallstone impacted in the duodenum. A diagnosis of Bouveret's syndrome was made. The management of this rare cause of gastric outlet obstruction is discussed.

## Background

Gallstones, in the majority of patients remain asymptomatic. The commonest clinical manifestation is biliary colic. Gallstone ileus occurs when a stone enters the intestinal tract via a cholecysto-enteric fistula. The authors present a case of Bouveret's syndrome, a rare complication of gallstone disease and rare cause of gastric outlet obstruction.

## Case presentation

**Figure 1:**
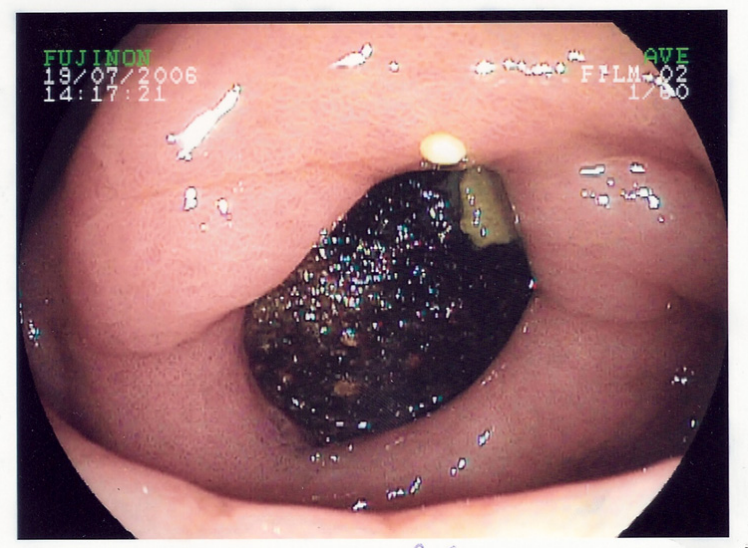
View at the pylorus, demonstrating a mass in the duodenal bulb.

**Figure 2:**
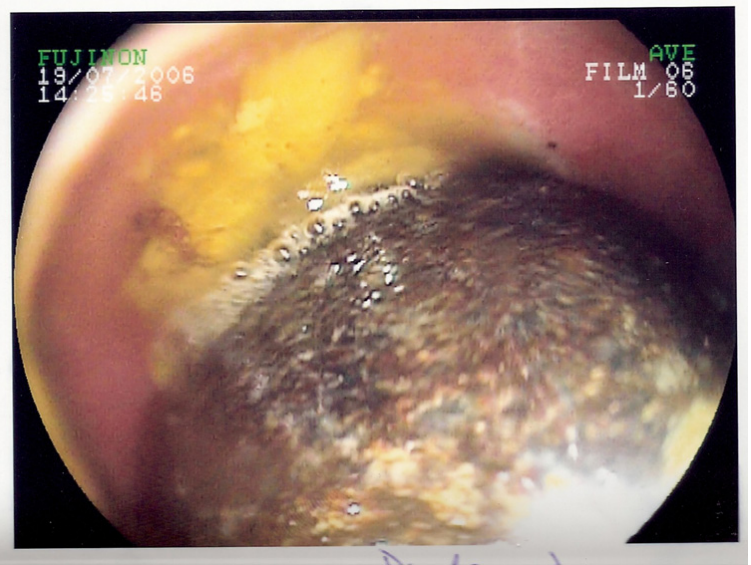
A large smooth mass in the first part of the duodenum with associated ulceration.

An 83 year old gentleman was admitted with a one week history of vomiting after eating and left-sided upper quadrant abdominal pain. There was no history of dysphagia or weights loss. The patient had suffered a similar episode the year previously which had resolved spontaneously. Abdominal examination was unremarkable. No succession splash was evident. A full blood count, liver function tests and urea and electrolytes were normal. No free air under the right hemi-diaphragm was noted on a chest radiograph. A plain abdominal film was negative for evidence of aerobilia or gallstones. A naso-gastric tube was inserted. The patient subsequently underwent an oesophago-gastro-duodenoscopy (OGD) to exclude possible mechanical obstruction. At OGD, a mass was noted beyond the pylorus (Figure 1). In the first part of the duodenum, the large smooth mass was seen occupying the whole lumen with ulceration of the visible surrounding mucosa (Figure 2). The mass was irretrievable endoscopically.

**Figure 3:**
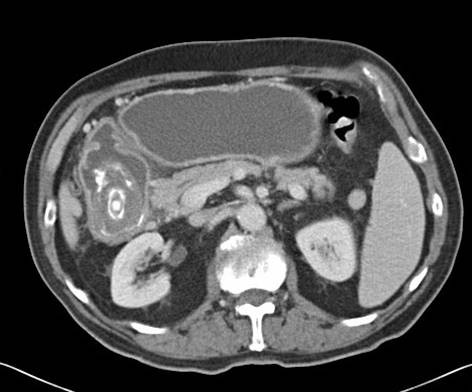
Abdominal CT demonstrating a calcified mass in duodenum.

Computed tomography (CT) of the abdomen demonstrated a large calcified mass in the first part of the duodenum (Figure 3). The patient underwent an open laparotomy where a cholecysto-duodenal fistula was found with a large gallstone impacted in the duodenum. No other synchronous gallstones were discovered. The gallstone was irretrievable and therefore a gastro-jejunostomy was performed. A diagnosis of Bouveret's syndrome was made.

**Figure 4:**
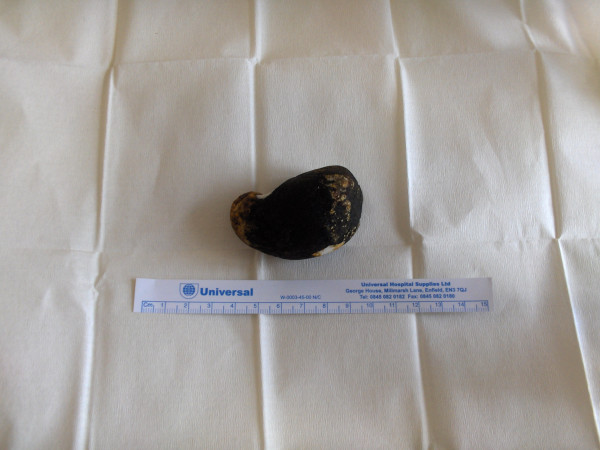
Removed gallstone.

Post operatively the patient continued to produce large gastric aspirates via a naso-gastric tube. A repeat OGD demonstrated that both afferent and efferent loops were patent. The large gallstone was noted once again but this time in the second part of the duodenum. The patient returned to theatre where this time the gallstone (Figure 4) was successfully milked into the distal jejunum and removed via an enterotomy. The patient made an uneventful recovery.

## Case discussion

Gallstone ileus is rare [1]. The majority of gallstones that enter the GI tract via a cholecysto-enteric fistula are passed spontaneously. Obstruction most commonly occurs in the terminal ileum (90%) and less often in the duodenum (3%) [2]. The differential diagnosis of gastric outlet obstruction includes diverticulae, foreign bodies, fibrotic ulcers and neoplasia Gastric outlet obstruction secondary to an impacted gallstone in the pyloric region or duodenal bulb is known as Bouveret's syndrome. More common in elderly women, Bouveret's syndrome presents with a non specific triad of epigastric pain, nausea and vomiting. Abdominal and chest radiographs should be performed looking for evidence of aerobilia, bowel obstruction and ectopic gallstones. Abdominal CT should also be performed. Typical findings on OGD include a dilated stomach and a hard non-fleshy mass at the obstruction [3].

Treatment options include endoscopic and surgical management. Endoscopic removal should always be attempted first, but lithotripsy and stone extraction is rarely successful [4]. Intracorporeal endoscopic electrohydraulic lithotripsy has been used successfully in the treatment of Bouveret's syndrome [5] Surgical options include enterotomy and removal of the stones (enterolithotomy), enterolithotomy plus cholecystectomy and repair of the fistula, or gastric bypass surgery. The decision to use minimal invasive surgery versus laparotomy should be made on an individual patient basis and operator experience. Fistula repair is unnecessary due to spontaneous closure especially if the cystic duct is patent and no residual stones are present. Post operative mortality rates are high, and may reflect the older subgroup of patients affected [6].

## Conclusion

The authors present a case of Bouveret's syndrome in an 83 year old gentleman. The diagnosis should be considered in patients with symptoms of gastric outlet obstruction with or without a history of gallstones or aerobilia and typical endoscopic findings of a dilated stomach and a hard non-fleshy mass at the obstruction.

## Competing interests

The author(s) declare that they have no competing interests.

## Authors' contributions

DJ, AV and TR were involved in writing of the case report. CF and AD were involved in the review and re-writing of the case report. All five authors were involved in the patient's care.
